# Bridging Tradition and Innovation: A Systematic Review and Bibliometric Analysis of Turkish Fermented Foods

**DOI:** 10.3390/foods14244324

**Published:** 2025-12-15

**Authors:** Özge Kahraman Ilıkkan, Elif Şeyma Bağdat, Remziye Yılmaz, Bülent Çetin, Ahmet Hilmi Çon, Hüseyin Erten, Mehmet Yekta Göksungur, Ömer Şimşek

**Affiliations:** 1 Food Quality Control and Analysis Programme, Department of Food Processing, Kahramankazan Vocational School, Başkent University, Ankara 06800, Türkiye; okilikkan@baskent.edu.tr; 2 Food Technology Programme, Department of Food Processing, Kahramankazan Vocational School, Başkent University, Ankara 06800, Türkiye; esbagdat@baskent.edu.tr; 3 International Food Biosafety and Biotechnology Research and Extension Center (IFBBC), Beytepe Campus, Hacettepe University, Ankara 06800, Türkiye; 4 Food Omics Laboratory, Department of Food Engineering, Beytepe Campus, Hacettepe University, Ankara 06800, Türkiye; 5 Department of Food Engineering, Beytepe Campus, Hacettepe University, Ankara 06800, Türkiye; 6 Faculty of Agriculture, Department of Food Engineering, Atatürk University, Erzurum 25030, Türkiye; bulent.cetin@atauni.edu.tr; 7 Department of Food Engineering, Faculty of Engineering, Ondokuz Mayıs University, Samsun 55270, Türkiye; ahmeth.con@omu.edu.tr; 8 Department of Food Engineering, Faculty of Engineering, Çukurova University, Adana 01250, Türkiye; herten@cu.edu.tr; 9 Department of Food Engineering, Faculty of Engineering, Ege University, Izmir 35040, Türkiye; yekta.goksungur@ege.edu.tr; 10 Department of Food Engineering, Faculty of Chemical and Metallurgical Engineering, Yıldız Technical University, Istanbul 34349, Türkiye; omers@yildiz.edu.tr

**Keywords:** Türkiye (Turkey), traditional fermented foods, fermentation, microbial terroir, functional foods

## Abstract

Background: Traditional fermented foods from Türkiye are integral components of the nation’s culinary heritage, reflecting a remarkable diversity shaped by local practices and ecosystems. These products embody region-specific microbial communities, often conceptualized as a “microbial terroir,” which influence their sensory qualities, nutritional value, and health-promoting properties. Methods: This study followed the PRISMA 2020 guidelines and included a systematic review and bibliometric analysis. A structured search was conducted in the Web of Science Core Collection (WoSCC) on 15 January 2025 using predefined keywords related to Turkish fermented foods and fermentation processes. Records were screened based on language (English or Turkish) and document type (articles, reviews, book chapters, and early access). A total of 1464 studies met the eligibility criteria, reflecting a 2.81% annual growth rate and a 12.7% international co-authorship rate. Bibliometric analysis was performed using the bibliometrix R package (RStudio 2024.12.1) and the biblioshiny interface. Results: The analysis revealed that the diversity of microbial consortia in Turkish fermented foods contributes to their distinctive characteristics, including enhanced nutritional profiles, probiotic potential, and food safety attributes. Emerging studies employing omics technologies—such as next-generation sequencing, metagenomics, and metabolomics—have expanded our understanding of fermentation ecosystems. Additionally, the growing integration of artificial intelligence supports predictive modeling and process optimization for advanced quality control. Conclusion: This synthesis highlights the significant technological, nutritional, and cultural value of Türkiye’s traditional fermented foods. Future directions should include omics-based translational research, indigenous starter culture development, and strengthened international collaborations to support sustainable and competitive functional food innovation.

## 1. Introduction

Türkiye’s traditional fermented foods show the close connections between culinary practices, environment, and microbial ecology, highlighting their potential for innovation and health applications. These products contribute not only to daily nutrition but also support health via beneficial microorganisms, which are inspired by localized environmental and cultural factors. The concept of “microbial terroir” has gained attraction in this context, describing how regional microbial communities influence the safety, flavor, and nutritional value of fermented products [1,2]. In an era marked by global food security challenges, these culturally grounded, nutrient-dense products offer affordable and sustainable options.

Scientific tools such as next-generation sequencing, metagenomics, and metabolomics have enhanced the resolution of microbial and biochemical profiling in recent years [3]. Integrative omics platforms have enabled a comprehensive understanding of fermentation ecosystems, illuminating both microbial structure and functional attributes [4,5]. Within this evolving landscape, fermentation extends various domains, from traditional spontaneous methods (e.g., kefir, boza, shalgam, tarhana, sourdough) to precision fermentation using engineered strains for targeted production of bioactives like enzymes or pigments [6,7]. These diverse approaches are unified by their potential for nutritional enhancement, food safety, and biotechnological innovation [8]. Furthermore, fermented products are increasingly seen as carriers of bioactive peptides and postbiotics, contributing to gut and immune health [9]. Despite the growing body of research, significant gaps persist: (i) mechanistic insights into strain–metabolite interactions, (ii) limited multi-omics integration for translational applications, and (iii) insufficient clinical validation of claimed health effects. These gaps highlight the need for a comprehensive synthesis that integrates current scientific advances with cultural heritage and innovation pathways.

Dairy-based fermentations, including yogurt, ayran, kefir, kurut, and koumiss, remain central to Türkiye’s fermented food portfolio. For instance, yogurt and ayran, produced via *Streptococcus thermophilus* and *Lactobacillus delbrueckii*, are widely studied for their probiotic benefits [10,11]. Kefir, with its symbiotic bacterial and yeast matrix, has been linked to immune and metabolic functions [12,13,14]. Traditional products such as kurut and koumiss offer insights into the historical continuity of dairy fermentation practices, including their therapeutic properties [15,16,17,18]. Microbiological and omics-based studies on cheeses such as tulum, herby, and white cheese further expand our understanding of microbial diversity and function in dairy fermentation [5,11,19,20,21,22].

Grain- and vegetable-based beverages like boza, shalgam, and hardaliye exhibit complex LAB-yeast dynamics that contribute to both sensory and biofunctional outcomes [23,24]. Alcoholic fermentations such as beer and wine have undergone both artisanal revival and industrial evolution in response to regulatory, cultural, and market influences [25,26,27,28]. Products such as gilaburu juice are also fermented to improve palatability and enhance antioxidant profiles [29].

Fermented meats such as pastırma and sucuk (soujouk), along with plant-based items like vinegar, pickles, shalgam, and olives, underscore the microbiological richness of Türkiye’s fermentation heritage [30,31,32]. Similarly, cereal-based fermentations such as tarhana and sourdough involve intricate interactions between lactic acid bacteria and yeasts, which shape flavor, texture, and nutritional properties while enhancing functional bioactive compounds [33,34,35].

Despite the abundance of descriptive studies, there remains a gap in mechanistic, translational, and clinical research, particularly around health outcomes and process optimization. Artificial intelligence (AI) is emerging as a novel tool for process modeling, safety monitoring, and precision fermentation [36,37]. Integrating omics-based strain profiling with AI-driven analytics offers a pathway toward elevating traditional fermented products into globally competitive, functional food platforms.

This review aims to synthesize the microbiological, nutritional, and cultural landscape of Türkiye’s traditional fermented foods while presenting a data-driven bibliometric analysis of the national research output between 2000 and 2025. This dual approach provides a foundation for identifying future directions in research, technology adoption, and heritage-based innovation within the broader context of sustainable food systems [3].

## 2. Traditional Fermented Foods in Türkiye

Key fermented products in Türkiye exhibit distinct microbial and regional signatures. Among these, cheese (Erzincan Tulum, Kars Gravyer, and Van Otlu (Herby) cheeses, etc.), yogurt, sucuk (soujouk), ayran, tarhana, kefir, shalgam, and boza are the most well-known and culturally embedded. Traditional region-specific cheeses host diverse LAB communities.

Yogurt is a conventional fermented dairy product that has been made in households or small communities in the Middle East for millennia. The presence of live lactic acid bacteria (LAB) and the high nutritional content of yogurt make it valuable. *Streptococcus thermophilus* and *Lactobacillus delbrueckii* subsp. *bulgaricus* are bacteria that are used in the fermentation of milk [10].

Ayran is the combination of salt, water, and milk or yogurt [38]. In commercial production, raw milk is pasteurized, fermented with starter cultures to produce buttermilk, and then cooled and stored [39]. The microbiota of Ayran consists mainly of *Streptococcus thermophilus* and *Lactobacillus delbrueckii* subsp. *bulgaricus*.

Tarhana, a cereal–yogurt-based product with remarkable geographical diversity, is produced through the fermentation of a cereal–yogurt mixture, which is subsequently sun-dried and milled into a stable powder. Tarhana is dominated by *Lactiplantibacillus plantarum*, *Pediococcus* spp., and *Saccharomyces cerevisiae* [40,41,42,43].

Kefir is prepared by inoculating milk with kefir grains containing a symbiotic consortium of bacteria and yeasts, allowing fermentation at ambient temperatures. It typically contains *Lactobacillus* spp., *Lactococcus* spp., *Leuconostoc* spp., *Acetobacter* spp., and yeasts, including *Kazachstania turicensis* [44,45].

Shalgam is a beverage prepared mostly from black carrots, bulgur flour, salt, and sourdough that is prized for its probiotic characteristics and nutritional value [46]. The manufacturing method includes lactic acid fermentation, which might take 10–12 days traditionally or 4–5 days commercially. Brine is characterized by the presence of *L. plantarum*, *Leuconostoc* spp., and *L. paracasei* [47,48,49].

Boza, on the other hand, is made by fermentation of maize, rice, wheat, millet, cracked wheat, and durum flour with *Lactobacillus fermentum*, *Limosilactobacillus brevis*, and *S. cerevisiae*, resulting in a mildly acidic, thick, and carbohydrate-rich beverage [24,50,51].

Among fermented meats, sucuk (soujouk) is a traditional dry fermented sausage that is extensively appreciated in Türkiye. It is usually made from sheep and/or beef meat with tail fat, curing agents such as nitrite/nitrate, and spices such as cumin, garlic, salt, black pepper, and red pepper. The mixture can be stuffed into natural casings made from beef small intestines or synthetic casings, fermented by indigenous microorganisms at 22–23 °C. Then, it is dried and aged for several weeks at room temperature and humidity (relative humidity of 45% to 80%) [52,53]. Lactic acid bacteria (LAB) and coagulase-negative staphylococci (CNS) play a key role in sucuk fermentation [54].

## 3. Cultural, Nutritional, and Economic Importance of Traditional Fermented Foods

**Figure 1:**
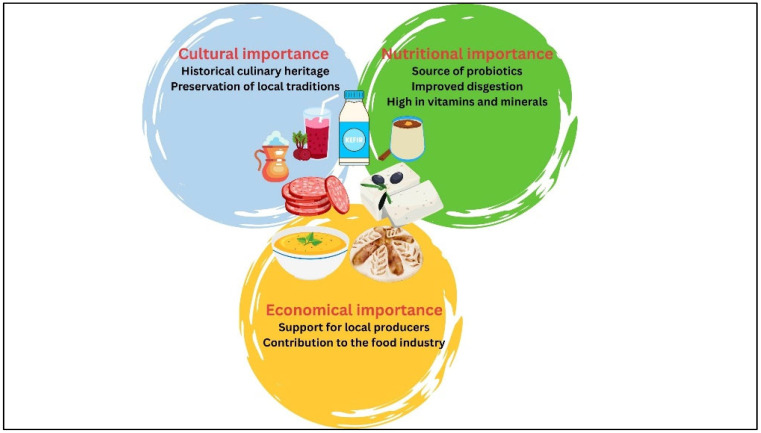
Cultural, nutritional, and economic importance of traditional fermented foods.

Traditional fermented foods in Türkiye are more than just dietary staples; they represent a deep-rooted connection of cultural heritage, nutritional practices, and economic livelihood. Passed down through generations, these foods are embedded in local customs, religious observances, seasonal rituals, and communal meals, reflecting both regional identity and historical continuity. An overview of selected traditional products and their defining characteristics is presented in Table 1, while Figure 1 highlights their interconnected cultural, nutritional, and economic dimensions.

Fermented products play a prominent role in social life and culinary culture all over the Türkiye. Items such as yogurt, ayran, tarhana, and boza are integral to traditional dietary patterns and are often prepared and consumed in the context of cultural events, family gatherings, or communal celebrations. The preparation of fermented foods, such as autumn pickling or tarhana drying, often involves multi-generational participation, helping to preserve both technical knowledge and community bonds. Notably, women play a pivotal role in these processes, with collective activities—particularly tarhana drying—serving as occasions for social interaction, knowledge exchange, and cooperative labor, thereby reinforcing community bonds and preserving culinary heritage. In many rural regions, especially in Anatolia, these food traditions remain central to local identity and seasonal rhythms.

Additionally, products like pastırma and sucuk are emblematic of Türkiye’s nomadic and Central Asian culinary roots, while others such as hardaliye, shalgam and gilaburu illustrate unique regional practices that have persisted over centuries. Beyond their historical and cultural dimensions, these products are also underpinned by place-specific microbial communities that guide the fermentation process. Microbial terroir plays a defining role in shaping the sensory and functional characteristics of Turkish fermented foods. Tarhana samples from Uşak, Tokat, Afyon, and Çorum demonstrate unique LAB compositions influenced by local cereal varieties, sun-drying conditions, and household fermentation vessels [43]. Eastern Anatolian kefir grains contain richer yeast diversity than western counterparts, reflecting differences in animal breeds, climate, and traditional grain propagation methods [55]. Likewise, Van Otlu (Herby) cheese, Erzurum Küflü cheese, and Kargı Tulum exhibit region-specific microbial communities shaped by milk microbiota, endemic herbs, and distinctive fermentation environments [56,57,58].

Nutritionally, fermented foods contribute significantly to dietary diversity and health. The fermentation process enhances digestibility, reduces anti-nutritional factors, and generates beneficial compounds such as organic acids, vitamins, enzymes, and bioactive peptides. For example:

- Yogurt, kefir, and ayran are rich sources of calcium, protein, and lactic acid bacteria, which probably promote gut microbiota balance and immune modulation;
- Sourdough breads and tarhana reduce phytates, thereby improving the absorption of minerals and overall protein digestibility;
- Fermented vegetables and pickles contain fiber, antioxidants, and vitamins such as C and K, supporting metabolic and cardiovascular health.

These nutritional contributions are particularly relevant in current contexts where lactose intolerance, gastrointestinal conditions, and non-communicable diseases are prevalent. Moreover, the probiotics and postbiotic compounds in fermented foods are increasingly recognized for their roles in immune regulation, mental health, and chronic disease prevention. However, the degree of scientific evidence supporting these health outcomes differs substantially across fermented products. The strength of evidence supporting health effects varies considerably across fermented foods. Kefir and yogurt show the strongest support through human clinical studies demonstrating improvements in gut microbiota modulation, lipid metabolism, and immune function [59]. Tarhana, boza, and sourdough display moderate evidence derived mainly from in vitro and animal models, indicating antimicrobial, antioxidant, and anti-inflammatory activities [60,61,62]. Hardaliye and gilaburu juice exhibit promising antioxidant potential but require well-designed clinical trials to validate health claims [29,63,64]. This categorization ensures accurate representation of evidence levels.

From an economic standpoint, traditional fermented foods contribute to livelihoods at the household, local, and national levels. Artisanal production sustains rural economies, particularly through the involvement of women and small-scale producers who engage in the making of yogurt, cheese, olives, and pickles. Local markets often serve as platforms for distributing these products, which also find growing demand in urban centers due to rising consumer interest in natural, minimally processed, and functional foods. Additionally, certain products, such as tarhana (11 varieties), hardaliye (1 variety), and various local cheeses (38 varieties), sourdough (17 varieties), are protected under geographical indication (GI) schemes, enhancing their economic value and branding appeal [65]. Türkiye possesses robust industrial and export capacity for fermented foods, supported by modern processing technologies, GI registrations, and expanding international markets.

At an industrial scale, Türkiye’s fermented food sector has become a major pillar of the agri-food economy. The domestic and export markets for yogurt, ayran, pastırma, sucuk, table olives, wine, and beer have expanded steadily, supported by both traditional know-how and modern manufacturing. Fermented milk product exports (Harmonized System, HS 0403) in 2023 were USD 26.8 million (OEC Report, https://oec.world/en/profile/country/tur (accessed on 1 September 2025)).

As Table 1 shows, GI registration has been granted to a broad range of products across multiple categories, demonstrating both the geographic diversity and commercial potential of Türkiye’s fermentation heritage. The emergence of gourmet, organic, and diaspora-oriented food channels has further opened avenues for international branding and gastronomic tourism.

Overall, traditional fermented foods in Türkiye place at the crossroads of culture, nutrition, and sustainable development. Their continued production, protection, and innovation not only preserve intangible cultural heritage but also provide scalable opportunities to improve health outcomes and foster rural resilience.

**Table 1:** List of some geographical indication of Turkish Fermented Foods (Turkish Patent and Trademark Office) (https://ci.turkpatent.gov.tr/ (accessed on 1 September 2025)), [65].

Fermented Food	Geographical Indication	City	Registration Numbers
Tarhana	Cide Tarhanası	Kastamonu	1380
	Kızılcık Tarhanası (Cornelian Cherry Tarhana)	Bolu- Kütahya	252
	Sıkma Tarhana	Amasya	1300
	Fethiye Tarhanası	Muğla	1484
	Şirin Tarhanası	Gaziantep	1078
	Gediz Tarhanası	Kütahya	1108
	Malatya Tarhanası	Malatya	1174
	Maraş Tarhanası	Kahramanmaraş	154
	Göce Tarhanası	Muğla	458
	Üzüm Tarhanası (Grape Tarhana)	Tokat	1612
	Uşak Tarhanası	Uşak	209
Ayran	Susurluk Ayranı	Balıkesir	238
Vinegar	Sirkencübin	Konya	G7
Boza	Velimeşe Bozası	Tekirdağ	721
Shalgam	Adana Şalgamı	Adana	489
	Tarsus Şalgamı	Mersin	84
Hardaliye	Kırklareli Hardaliyesi	Kırklareli	278
Olive	Akhisar Uslu Zeytini	Manisa	165
	Akhisar Domat Zeytini	Manisa	166
	Yamalak Sarısı Zeytini	Aydın	732
	Memecik Zeytini	Aydın	749
	Edremit Körfezi Yeşil Çizik Zeytini	Balıkesir	189
	Edincik Zeytini	Balıkesir	1556
	Büyükbelen Tekir Zeytini	Manisa	1611
	Gemlik Zeytini	Bursa	76
	Halhalı Zeytini	Hatay	1353
	Karamürsel Samanlı Zeytini	Kocaeli	1622
	Milas Ekşili Zeytini	Muğla	1691
	Milas Yağlı Zeytini	Muğla	446
	Milas Çekişke Zeytini	Muğla	1109
	Tarsus Sarıulak Zeytini	Mersin	345
Pickle	Acur-Biber Turşusu	Gaziantep	1057
	Midyat Turşusu/Midyat Acur Turşusu	Mardin	1131
	Çorti Turşusu	Muş	294
	Orhangazi Gedelek Turşusu	Bursa	249
	Taflan Turşusu	Ordu	1102
	Yayla Pancarı Turşusu	Ordu	269
	Kapari Turşusu	Osmancık	1636
	Reşadiye Kırmızı Pezik Turşusu	Tokat	1455
	Pezik Turşusu	Sivas	492
	İskilip Turşusu	Çorum	131
	Çubuk Turşusu	Ankara	99
Sucuk	Bez Sucuk	Tokat	990
Gilaburu	Akkışla Gilaburusu	Kayseri	669
	Bünyan Gilaburusu	Kayseri	389
	Gemerek Gilaburusu	Sivas	241
Cheese	Carra Peyniri	Hatay	679
	Sıkma Peyniri	Gaziantep	356
	Atlantı Dededağ Tulum Peyniri	Konya	1327
	Ayvalık Kelle Peyniri	Balıkesir	1664
	Tulum Peyniri	Ağrı	1565
	Bergama Tulum Peyniri	İzmir	1597
	Örgü Peyniri	Diyarbakır	170
	Beyaz Peyniri	Edirne	93
	Elbistan Kelle Peyniri	Kahramanmaraş	1500
	Tulum Peyniri	Erzincan	30
	Civil Peyniri	Erzurum	116
	Küflü Civil Peyniri	Erzurum	164
	Ezine Peyniri	Çanakkale	86
	Deleme Peyniri	Gümüşhane	694
	Hanak Tel Peyniri	Ardahan	1563
	Divle Obruğu Tulum Peyniri	Karaman	270
	Kargı Tulum Peyniri	Çorum	933
	Gravyer Peyniri	Kars	1640
	Kepsut Bükdere Küflü Katık Peyniri	Balıkesir	1683
	Eski Kaşar Peyniri	Kırklareli	1408
	Beyaz Peyniri	Kırklareli	636
	Manyas Kelle Peynir	Balıkesir	628
	Malatya Peyniri	Malatya	1164
	Malkara Eski Kaşar Peyniri	Tekirdağ	261
	Maraş Parmak/Sıkma Peynir	Kahramanmaraş	727
	Mengen Peyniri	Bolu	1482
	Pınarbaşı Uzunyayla Çerkes Peyniri	Kayseri	724
	Sakarya Abhaz (Abaza) Peyniri	Sakarya	746
	Savaştepe Mihaliç Kelle Peyniri	Balıkesir	1405
	Talas Çörek(Çömlek Peyniri	Kayseri	1560
	Urfa Peyniri/Şanlıurfa Peyniri	Urfa	807
	Vakfıkebir Külek Peyniri	Trabzon	764
	Van Otlu (Herby) Peyniri	Van	405
	Yozgat Çanak Peyniri	Yozgat	281
	Çankırı Küpecik Peyniri	Çankırı	907
	Çayeli Koloti Peyniri	Rize	1199
	İvrindi Kelle Peynir	Balıkesir	1025
	İzmir Tulum Peyniri	İzmir	1006
Pastırma	Afyon Pastırması	Afyon	73
	Ankara Erkeç Pastırması	Ankara	408
	Erzurum Pastırması	Erzurum	1311
	Kastamonu Pastırması	Kastamonu	711
	Kayseri Pastırması	Kayseri	36
	Sivas Pastırması	Sivas	1129
Sourdough	Afyonkarahisar Ak Pide	Afyon	1265
	Mamak Kutludüğün Ekşi Maya Ekmeği	Ankara	827
	Kürtün Araköy Ekmeği	Gümüşhane	449
	Kalecik Ekmeği	Ankara	559
	Gümüşhane Ekmeği	Gümüşhane	221
	Tokat Ekmeği	Tokat	896
	Vakfıkebir Ekmeği	Trabzon	372
	Çavuşlu Ekmeği	Giresun	623
	Bolu Patates Ekmeği Bolu	Bolu	544
	Çöven Ekmeği	Bartın	1045
	Afyonkarahisar Patates Ekmeğ	Afyon	371
	Alaşehir Ekmeği	Manisa	1119
	Cihanbeyli Gömeç Ekmeği	Konya	1241
	Gelveri Ekmeği	Aksaray	1040
	Malatya Ekmeği	Malatya	1127
	Erzurum Hasankale Lavaşı	Erzurum	1020
	Erzurum Acem Ekmeği	Erzurum	1365

## 4. Bibliometric Analysis

While the preceding sections have demonstrated the cultural, nutritional, and economic relevance of Türkiye’s traditional fermented foods, this section provides a quantitative and structured bibliometric overview, clarifying the evolving research landscape and international collaborations in this domain. This review was conducted in accordance with the PRISMA 2020 guidelines. The PRISMA flow diagram and the completed PRISMA 2020 Checklist are provided in the Supplementary Materials (Table S1). The Web of Science Core Collection (WoSCC) was searched on 15 January 2025. The search strategy was framed around publications related to both traditional and contemporary fermentation practices in Türkiye. A combination of primary keywords was utilized, including specific fermented products and fermentation-related terms: ‘yogurt’ OR ‘vinegar’ OR ‘Ayran’ OR ‘boza’ OR ‘shalgam’ OR ‘kefir’ OR ‘kurut’ OR ‘kımız’ OR ‘kimiz’ OR ‘Koumiss’ OR ‘hardaliye’ OR ‘tarhana’ OR ‘olive’ OR ‘pickle’ OR ‘gilaburu’ OR ‘butter’ OR ‘fermented sausage’ OR ‘sourdough’ OR ‘beer’ OR ‘wine’ OR ‘pastırma’ OR ‘soujouk’ OR ‘cheese’ OR ‘sucuk’ OR ‘water kefir’ AND ‘fermentation’ OR ‘fermented’ AND ‘Turkey’ OR ‘Turkish’ OR ‘Türkiye’. The bibliometric search strategy used combined controlled vocabulary and free-text keywords (‘fermentation’, ‘fermented’, ‘traditional foods’, ‘Türkiye’, ‘Turkish’) with Boolean operators to maximize coverage. Records were retrieved exclusively from Web of Science Core Collection to ensure high-quality indexing. All 1501 initial records were screened using predefined inclusion criteria: (i) English or Turkish language; (ii) categorized as research articles, reviews, book chapters, or early access publications; (iii) explicit relevance to fermentation science or fermented food products. Exclusion criteria also eliminated irrelevant domains such as biofuel fermentation, pharmaceutical fermentation, and industrial enzyme production. This structured approach increases the reliability and reproducibility of the mapping.

**Figure 2:**
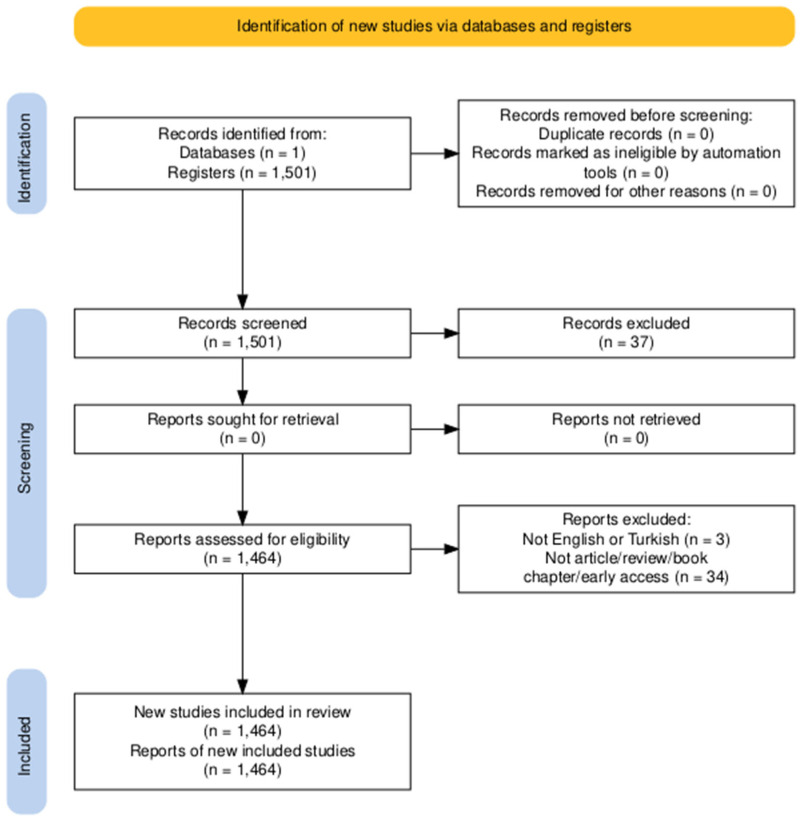
PRISMA flow chart for including and excluding studies.

All retrieved records were screened using database filters and manual relevance evaluation. Duplicate records were automatically removed by the database *(n* = 0). Two researchers independently reviewed titles and abstracts to determine inclusion, and discrepancies were resolved by consensus. A total of 1464 items were retained for analysis. The publication window was defined from January 2000 to January 2025, offering a broad lens on historical and current research patterns. The data were extracted directly from the WoSCC database and processed using the R package bibliometrix and biblioshiny interface, ensuring standardized extraction of authors, institutions, keywords, citations, and document types. A PRISMA flow chart summarizing this filtering process is illustrated in Figure 2. This methodology provides comprehensive coverage of Türkiye’s fermentation literature, laying a strong foundation for the bibliometric exploration and insights into traditional and modern practices.

**Figure 3:**
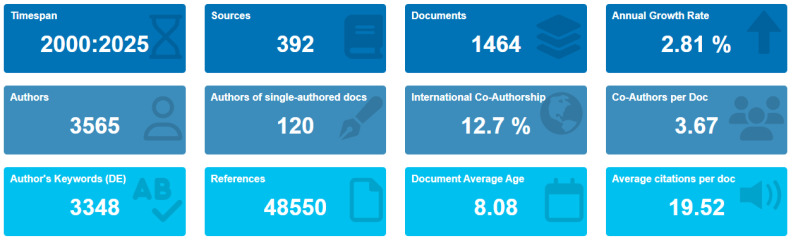
Overview of the publications found. The growth rate of the topic is observed to be 2.81%, indicating a significant increase in interest and research in recent years. The average age of the documents is 8 years, suggesting that the topic is relatively recent and still evolving. The international co-authorship rate was 12.7%. This highlights the ongoing development and expanding scope of fermentation research, particularly in the context of traditional and modern practices in Türkiye.

The annual growth rate of publications in this domain was calculated at 2.81%, indicating a steady rise in interest and scholarly contributions. The mean age of documents is 8 years, reflecting the relative recentness and development of the relevant field. The international co-authorship rate was determined to be 12.7%, highlighting a moderate level of global collaboration in fermentation research specific to Türkiye (Figure 3).

**Figure 4:**
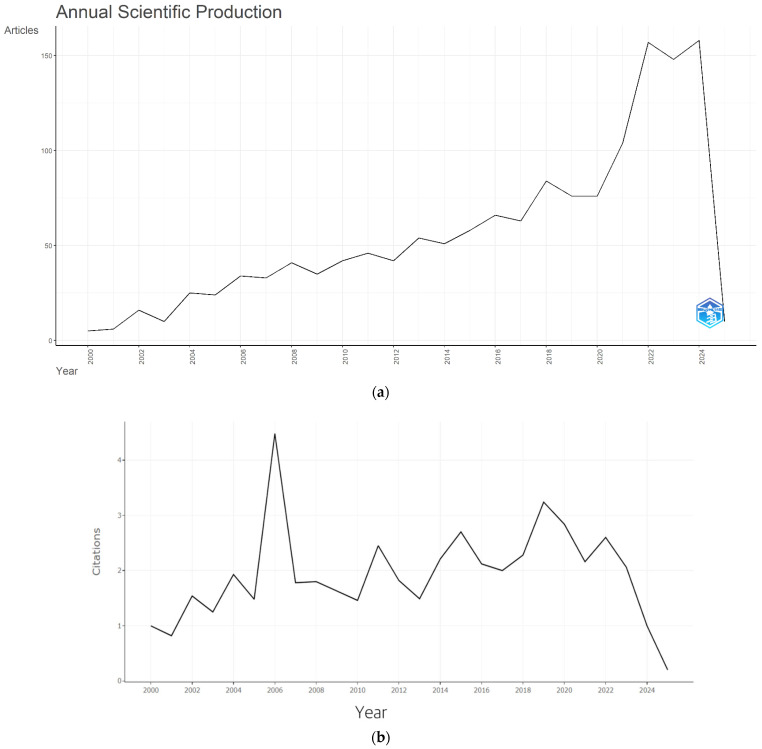
Annual publication growth rate (**a**) and average citations per year (**b**).

The annual trajectory of publications follows a logarithmic trend, reflecting both the growing scientific interest and the expanding methodological toolkit in fermentation research, with 158 papers published in 2024 and an additional 10 appearing in early 2025 (Figure 4a). The pronounced acceleration observed after 2020 coincides with the widespread adoption of omics approaches, which have enabled deeper insights into microbial community structures, metabolic pathways, and functional properties of fermented foods. This surge not only underscores the increasing recognition of fermentation research in food science and technology but also suggests a sustained momentum for future studies that integrate advanced molecular techniques with traditional food systems.

Average citation analysis in Figure 4b suggests that fermentation research began to generate noticeable academic impact between 2000 and 2006. The mid-2000s saw an emphasis on health benefits of fermented foods, probably due to the growing popularity of probiotic and functional food research. Although some fluctuations in citation rates are observed post-2010, a general upward trend persisted until 2020. The relative decline in citations after 2020 is primarily due to recency, as more recent publications have not yet had sufficient time to accumulate citations. Additional contributing factors may include the rapid increase in publication volume, which disperses citations across a larger number of articles, the temporary research shifts induced by the COVID-19 pandemic, and the initially narrower visibility of highly technical or niche studies employing omics approaches.

### 4.1. Author’s Keywords and Research Connections

**Figure 5:**
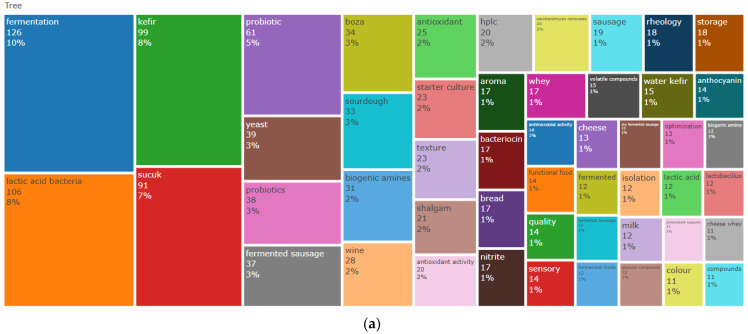

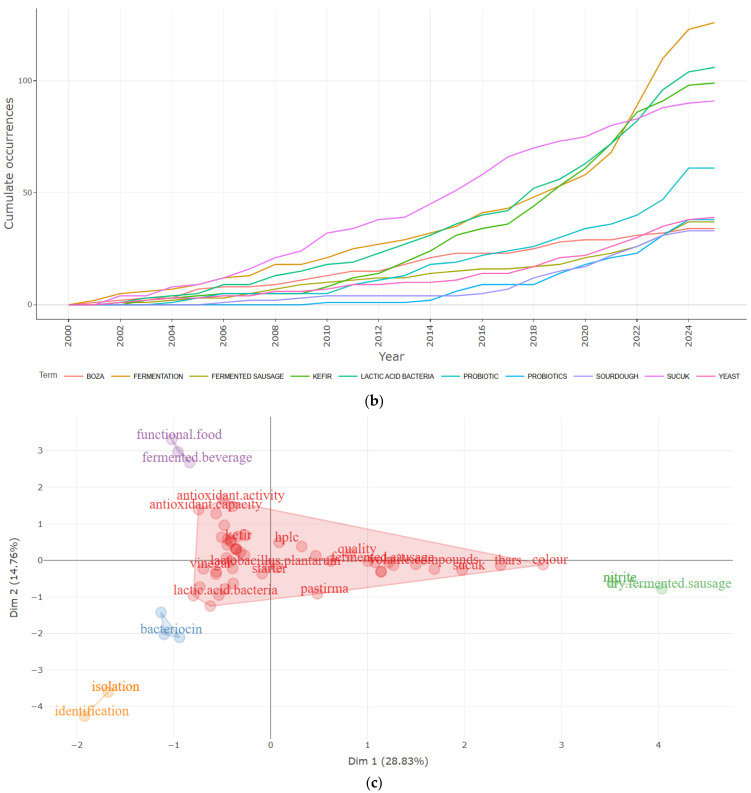
Percentage distribution of keywords selected by authors in the publications (**a**); yearly frequency increase in keywords (**b**); factorial map (multiple correspondence analysis) (**c**).

To evaluate how cultural and scientific dimensions are manifested in academic outputs, this section explores keyword distributions, thematic trends, and interconnections among research topics. Figure 5a presents the relative frequency and dispersion of keywords chosen by authors across the bibliometric dataset. ‘Fermentation’ emerged as the most prevalent term, underscoring its foundational role in the field, followed by high-frequency terms such as ‘lactic acid bacteria’, ‘kefir’, and ‘sucuk’.

Figure 5b tracks the temporal rise in selected keyword frequencies from 2000 to 2025. Each trajectory illustrates how interest in specific themes, ranging from traditional to modern fermentation practices, has evolved, both within Türkiye and globally. Notably, the keyword ‘Fermentation’ (represented by the brown line) has shown a consistently increasing trend, reinforcing its centrality and anticipated prominence in future investigations. This trend likely reflects technological advancements in genomics, transcriptomics, proteomics, and artificial intelligence.

Probiotics have also gained momentum, reflecting growing interest in the health-promoting aspects of fermented foods. Figure 5c depicts a Multiple Correspondence Analysis (MCA) of author keywords, with axes representing Dimension 1 (28.83%) and Dimension 2 (14.76%), resulting in five distinct thematic clusters.

The red cluster, covering a broad conceptual space, includes terms such as ‘*Lactiplantibacillus plantarum*’, ‘starter’, ‘kefir’, ‘pastırma’, ‘antioxidant activity’, ‘sucuk’, ‘tbars’, and ‘quality’, which are tightly interlinked and contribute significantly to overall variation. This cluster likely reflects research focused on core quality and functional parameters of fermented foods, such as oxidative stability, color changes, and starter culture selection. Traditional Turkish fermented foods serve as a rich microbial reservoir, whose genetic diversity facilitates the discovery of novel genes and metabolic traits, thereby supporting the development of industrial microorganisms capable of producing enzymes, bioactive proteins, and other functional biomolecules. While these functional properties suggest promising health-related benefits, it is important to note that many findings remain limited to in vitro or animal models. Although several studies report potential antioxidant, immunomodulatory, and antimicrobial effects, these claims require further clinical validation before broader health implications can be established.

The blue cluster centers around ‘bacteriocin’, which appears in relative isolation. This spatial separation suggests that bacteriocin-related studies diverge from the main axis of antioxidant and meat-fermentation-focused themes. Notably, several recent studies have successfully isolated and characterized novel bacteriocins from traditional Turkish fermented foods such as tarhana and sourdough, highlighting their untapped potential as natural antimicrobial agents and reinforcing the value of these foods as reservoirs of functional microbial diversity [33,34]. Meanwhile, the green cluster, characterized by ‘nitrite’ and ‘dry fermented sausage’, indicates an association with meat-based fermentations, albeit distinct from antioxidant or antimicrobial mechanisms.

The purple cluster links ‘functional food’ with ‘fermented beverage’, reflecting contemporary categorization trends where beverages like kefir and boza are recognized as functional foods. Lastly, the orange cluster, composed of terms like ‘isolation’ and ‘identification’, emphasizes microbial discovery and characterization, foundational to both applied and basic fermentation research.

### 4.2. Thematic Map and Trend Topics

**Figure 6:**
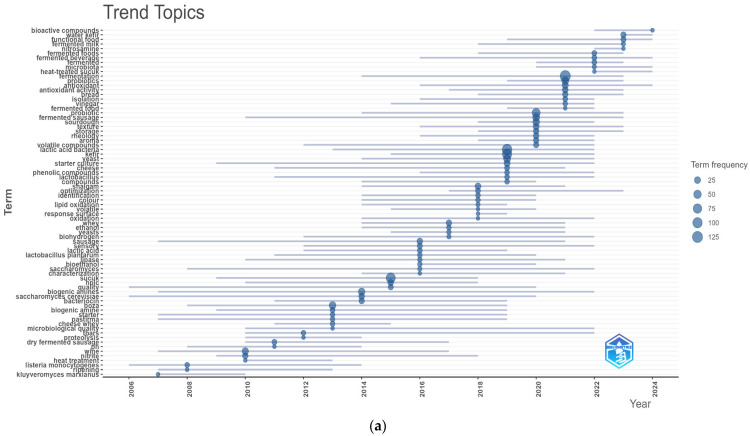

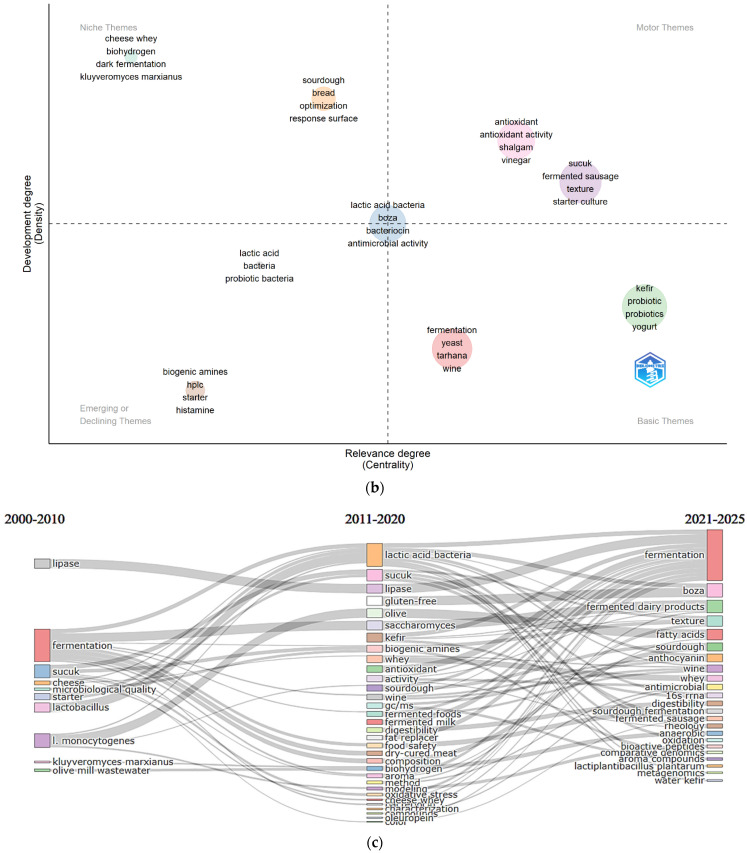
Thematic map (**a**); trend topics (**b**); and thematic evolution map (Sankey diagram) (**c**).

The thematic map offers insights into how key research themes align with technological, functional, and health-related attributes of fermented foods. In Figure 6a, motor themes are defined by high centrality and strong development, reflecting areas that are both well-established and influential within the field. These include ‘antioxidant activity’, ‘shalgam’, ‘sucuk’, ‘fermented sausage’, ‘starter culture’, and ‘vinegar’. Their prominence signifies that these topics are not only widely studied but also critical for advancing fermentation research in Türkiye.

The term ‘antioxidant activity’ has emerged as a principal focus in evaluating the health impacts of fermented products. ‘Shalgam’ (a lactic acid fermented beverage based on black carrot), a culturally significant beverage, has gained attention due to its potential probiotic and antimicrobial effects. Motor themes are both current and conceptually robust, positioning them as strategic focal points for future investigations.

Basic themes, while central to the field, display lower developmental density, indicating maturity or a decline in novelty. Terms such as ‘kefir’, ‘probiotics’, and ‘yoghurt’ fall into this quadrant. These keywords represent foundational knowledge in fermented food research and continue to influence ongoing studies despite not being newly emergent.

The emerging or declining themes quadrant houses topics with lower centrality and developmental intensity. This includes terms like ‘biogenic amines’, ‘HPLC’, ‘histamine’, ‘lactic acid bacteria’, ‘probiotic bacteria’, ‘boza’, ‘antimicrobial activity’, ‘sourdough’, ‘bread’, ‘optimization’, ‘response surface’, ‘fermentation’, ‘yeast’, ‘tarhana’, and ‘wine’. Some of these, like ‘boza’ and ‘tarhana’, may represent recently renewed interest, possibly driven by new methodological approaches. Conversely, ‘biogenic amines’ and ‘HPLC’ may reflect diminishing research interest, potentially due to the field’s pivot from toxicity concerns to probiotic functionality.

Certain terms in this quadrant, such as ‘lactic acid bacteria’ and ‘antimicrobial activity’, remain highly relevant but may have become conceptually merged with broader themes like starter culture development. Keywords such as ‘optimization’ and ‘response surface’ are likely retained for experimental design applications, suggesting that while methodologically significant, they maintain a niche role.

Niche themes, including ‘cheese whey’, ‘biohydrogen’, ‘dark fermentation’, and ‘*Kluyveromyces marxianus*’, reflect specialized but well-developed research areas. These topics may hold high advanced but limited general applicability, serving as critical knowledge hubs within their subfields.

In Figure 6b, the Trend Topics graph tracks keyword usage across defined timeframes. Between 2004 and 2010, research activity was sparse, with only ‘lactic acid bacteria’ showing a minor rise. A significant shift occurs during 2015–2020, where terms like ‘probiotics’, ‘fermented milk’, and ‘bioactive compounds’ gain prominence. This upward trend continues from 2020 to 2024, with ‘bioactive compounds’ and ‘fermented foods’ peaking in 2024 (~125 citations each), reflecting growing interest in health-associated attributes of fermented products.

Similarly, ‘lactic acid bacteria’ remained a central focus during this period, reinforced by advancements in omics technologies such as next-generation sequencing and metagenomics. These metagenomic approaches have not only illuminated the fermentation microbiota of traditional foods but also revealed how fermentation conditions shape microbial community assembly. For instance, comparative studies on tarhana have demonstrated notable differences in microbiota composition between household-scale and industrial production, highlighting the influence of production scale and environmental factors on fermentation outcomes [43]. The increasing integration of the term ‘microbiota’ into fermentation studies further boosted attention on ‘probiotics’, which reached ~100 citations between 2020–2024.

Keywords like ‘fermentation’ and ‘bioactive compounds’ sustained moderate popularity (75–100 citations), while terms such as ‘dry fermentation’, ‘pastirma quality’, and ‘biogenic amine’ experienced stagnation, indicating lesser research momentum. These observations correlate with keyword frequencies shown in Figure 5a, suggesting a reorientation toward health, sustainability, and innovation in fermentation studies.

Figure 6c, a Sankey diagram, illustrates the evolution of fermentation research themes in Türkiye over three time periods. From 2000 to 2010, traditional fermentation practices were subjected to basic scientific inquiry, with emerging concerns around food safety (e.g., control of *Listeria monocytogenes*) and microbial ecology. Though sustainability topics, like olive oil waste valorization, began to surface, the phase was dominated by descriptive microbiological studies.

During 2011–2020, research transitioned into a convergence phase, incorporating biotechnology, health science, and consumer trends. Traditional products such as yogurt, kefir, dry sausage, sourdough, and wine were studied in relation to probiotics, antioxidants, and food safety innovations. Attention also turned to biopreservation, gluten-free adaptations, and starter culture optimization, indicating a more applied and functional research orientation.

The period 2021–2025 is characterized by cutting-edge tools and translational science. Traditional fermented products are investigated using metabolomics, amplicon-based metagenomics, and advanced functional profiling. Topics such as bioactive metabolite quantification and microbiota characterization dominate this phase. The widespread application of 16S rRNA sequencing, microbial community mapping, and health claims validation underscores a paradigm shift toward precision fermentation and evidence-based functional food design.

Throughout all periods, health-related themes remained pivotal. In the early phase, the focus was on safety (e.g., pathogen detection); in the middle phase, on functional benefits (e.g., antioxidants and probiotics); and in the modern phase, on molecular and metabolic evidence supporting health claims. Environmental considerations, such as waste valorization, persisted as cross-cutting elements. This evolution affirms how Türkiye’s fermentation heritage has transitioned into a multidisciplinary scientific landscape, offering valuable insights for global innovation, health, and sustainability.

**Figure 7:**
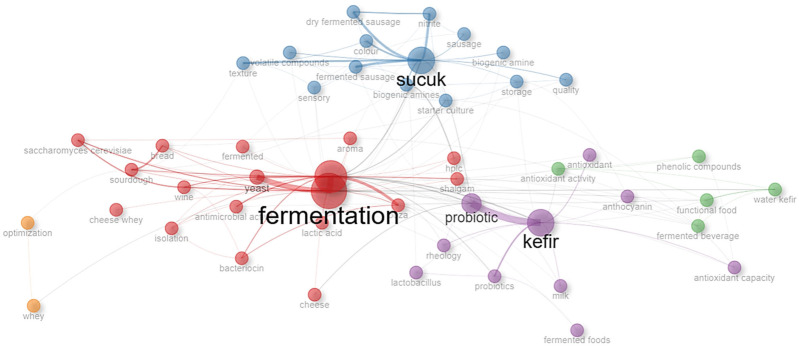
Keyword co-occurrence network and thematic clusters in fermentation research. This figure presents the relationship network and clusters of keywords used in fermentation research.

Figure 7 illustrates four major thematic clusters in the field: (1) dairy-based fermented foods and probiotic research; (2) cereal- and vegetable-based fermentations linked to nutritional and microbial diversity studies; (3) safety-oriented research addressing biogenic amines and fermentation control; and (4) emerging omics-driven approaches. These clusters reflect both historical research strengths and new scientific trajectories, thereby supporting the interpretation of fermentation as a multidisciplinary field in Türkiye. Node size represents the frequency of keyword occurrence, while edge thickness indicates the strength of co-occurrence between terms. Distinct color clusters highlight thematic research areas: (i) general fermentation and yeast-related studies (red), (ii) fermented meat products, particularly sucuk and biogenic amines (blue), (iii) kefir, probiotics, and functional dairy research (purple), and (iv) antioxidant- and phenolic compound-focused studies, including water kefir (green). The network reflects the interconnected structure of fermentation science and emerging research hotspots within the field.

### 4.3. International Collaborations on Turkish Fermented Foods

**Figure 8:**
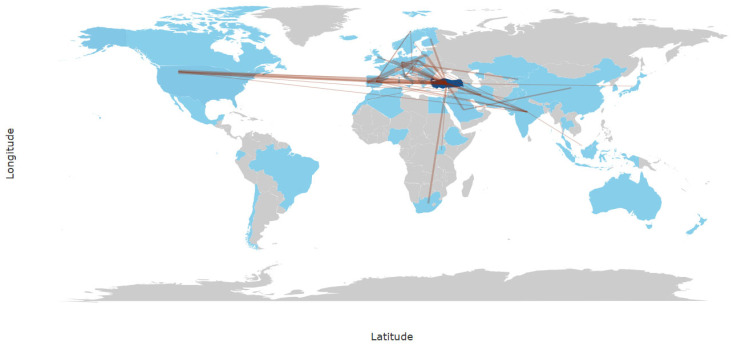
International collaboration and countries studying Turkish fermented products.

Understanding Türkiye’s positioning within the global fermentation research ecosystem is essential for recognizing how national expertise integrates with international scientific collaborations. The collaboration networks in Figure 8 underscore Türkiye’s growing integration into global fermentation research, with strong partnerships observed with the United States, Germany, Japan, and EU-based institutions. These partnerships are particularly prominent in kefir research, cheese microbiology, and culture-independent microbial analyses, reflecting how traditional practices are increasingly becoming subjects of cross-border academic and technological exchange.

The network map or diagram highlights key countries participating in studies of Turkish fermented foods, thereby positioning Türkiye as both a knowledge hub and research partner in global fermentation discourse. These international connections represent pathways through which microbial diversity, artisanal knowledge, and emerging biotechnologies are shared and expanded upon.

**Figure 9:**
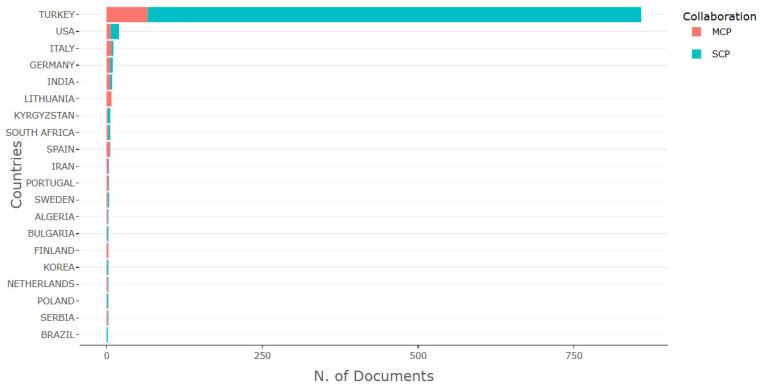
Publications by countries on the topic, including single and collaborative publications. SCP: Single country publication. MCP: Multi-country publication.

Figure 9 categorizes the publication data according to Single Country Publications (SCP) and Multiple Country Publications (MCP). This classification allows for a comparative analysis of independent versus collaborative scholarly outputs related to Turkish fermented foods. As expected, Türkiye leads with the highest SCP count, underlining a strong domestic research infrastructure sustained by universities, food technology departments, and public research institutions.

Nonetheless, a notable proportion of MCPs indicates that Türkiye is also actively engaging in international partnerships, involving shared research agendas, microbial repositories, biotechnological tools, and joint publications. These collaborations enable a broader scientific evaluation of traditional practices, enhancing methodological rigor and global relevance.

Interestingly, the downward trend in biogenic amine research, as depicted earlier, may signify a broader pivot in global fermentation research—from toxicity surveillance toward probiotic and functional innovations. The decline also parallels a growing emphasis on health claims validation and translational fermentation science, suggesting that collaborative efforts now prioritize contemporary challenges and opportunities.

Despite this, the limited representation of international joint works in certain regions, particularly in Asia and parts of Europe, highlights an untapped potential for Türkiye to extend its research diplomacy. As shown in Figure 8, fostering deeper partnerships, especially in regions sharing similar fermentation traditions, could enable cross-cultural and transregional studies that enrich both scientific and sociocultural perspectives.

In conclusion, the bibliometric analysis confirms that Türkiye’s rich heritage in fermentation has evolved into a scientifically vibrant and internationally relevant domain. However, expanding global collaborations, particularly in the context of functional food development, omics applications, and sustainability, remains a critical next step. By doing so, Türkiye can further translate its local expertise into global innovations, anchoring its traditional food systems within cutting-edge scientific dialogs.

## 5. Future Perspectives

Building on the cultural, nutritional, economic, and bibliometric insights outlined earlier, this section explores how emerging scientific approaches can shape the future of Türkiye’s fermented foods. The fermentation field in Türkiye is poised for a major transformation fueled by innovations in omics sciences and artificial intelligence (AI). These tools are reshaping our understanding and development of traditional Turkish fermented products like boza, kefir, tarhana, and shalgam. Omics-based platforms, particularly genomics, metabolomics, and proteomics, offer an in-depth view into the microbial ecology, bioactive compound profiles, and functional benefits of these products. Meanwhile, the integration of AI into fermentation research is facilitating predictive modeling, quality enhancement, and health impact forecasting. By harmonizing deep-rooted traditions with advanced scientific tools, these developments can position Türkiye at the forefront of global functional food innovation.

Future research should prioritize the development of indigenous starter cultures, omics-driven characterization of microbial metabolite networks, standardized fermentation protocols, and the clinical validation of functional claims. Integrating AI-supported safety systems—particularly for predicting biogenic amines—may further enhance product quality and global competitiveness.

### 5.1. The Role of Omics Technologies in Turkish Fermented Products Research

Omics methodologies allow for holistic insights into the biological complexity of fermented foods, uncovering both beneficial attributes and safety considerations. Bibliometric findings confirm that metagenomics currently dominates Turkish fermented food research, with products such as tarhana, kefir, vinegar, and cheese frequently investigated.

The microbial diversity of geographically protected Uşak tarhana, for example, was explored using next-generation sequencing, revealing climate-associated microbial variations [43,66]. Similarly, kefir grains [44,67], shalgam [68], and vinegar microbiota [69] have been extensively characterized. Cheese varieties such as Tulum and Otlu (Herby) peynir are among the most deeply studied, reflecting their cultural and commercial importance.

Despite this progress, research utilizing other omics layers—especially proteomics, transcriptomics, and metabolomics—remains scarce. Peptide release dynamics during kefir fermentation have only recently been assessed [70], and metabolite profiling of tarhana is still limited [66]. A wider adoption of these technologies could uncover enzyme profiles improving texture, or trace bioactive or harmful metabolites, advancing both safety and quality. As omics integration deepens, novel functional ingredients may be discovered, enhancing both domestic value and export potential.

Significant gaps remain in multi-omics and metagenomic research for several culturally important products such as hardaliye, gilaburu, Hatay Carra cheese, Kargı Tulum, homemade pickles, and sourdoughs from Eastern and Southeastern Anatolia. Limited genomic data restricts the ability to map microbial–metabolite interactions and hinders efforts to develop standardized starter cultures or assess probiotic and postbiotic potential. Expanded, high-resolution omics studies are therefore essential for future innovation. In addition, the lack of standardized genomic datasets across regional product categories, insufficient multi-omics approaches to resolve microbial–metabolite networks, and limited clinical validation for health-related claims continue to constrain translational applications. Developing indigenous starter cultures based on well-characterized strains and integrating AI-based safety monitoring (e.g., prediction of biogenic amines) represent key priorities for innovation.

### 5.2. Artificial Intelligence (AI) in Fermented Foods

AI is increasingly being applied in food sciences for its capabilities in automated prediction, pattern recognition, and complex process optimization [71,72]. In the context of fermentation, tools such as decision trees, random forest (RF), support vector machines (SVM), and k-nearest neighbor (kNN) algorithms have been utilized for microbial control and sensory optimization [72,73,74]. E-nose technologies, simulating human olfaction, have also been used to classify volatile compounds and aroma profiles in fermented foods [75].

A notable example is the use of an adaptive neuro-fuzzy inference system (ANFIS) for optimizing ultrasound parameters in Gilaburu juice processing [76]. Although applications in Türkiye are still emerging, AI holds considerable potential to customize fermentation protocols, minimize variability, and enhance product consistency. Priority areas include starter culture optimization for traditional products, predictive shelf-life modeling, and omics-AI integration for data-driven quality control. Further, policy development to support AI applications among artisanal producers would enhance both standardization and export readiness.

## 6. Conclusions

Based on bibliometric outcomes, Tarhana and Kefir clearly emerge as flagship candidates for bridging traditional knowledge with modern functional food innovation. Türkiye’s traditional fermentation heritage reflects a compelling synergy of deep-rooted cultural practices, nutritional richness, and growing scientific sophistication. This study demonstrates how traditional methods have evolved from local artisanal knowledge into a vibrant field enriched by metagenomics, multi-omics platforms, and AI-driven tools.

Türkiye’s capacity to unify ancient fermentation wisdom with emerging scientific strategies offers a unique opportunity for global leadership in sustainable food systems. To harness this potential, future efforts should focus on expanding international partnerships, fostering omics-based translational studies, and strengthening regulatory infrastructures. Exportation of innovative fermented foods requires full compliance with the Turkish Food Codex, validated safety specifications, and—when applicable—the Novel Food Regulation (EU 2015/2283). Multi-omics and AI-supported safety tools enhance traceability and regulatory robustness.

While home fermentation contributes to cultural preservation and reduced packaging waste, industrial-scale production ensures microbial safety, standardization, and accessibility. A hybrid model that integrates traditional household practices with precision fermentation represents the most sustainable pathway forward. This approach will ensure the continued relevance, safety, and competitiveness of Turkish fermented foods worldwide.

By presenting an integrated overview of tradition, innovation, and scientific opportunity, this review aims to catalyze interdisciplinary efforts that sustain and elevate the cultural and functional value of Türkiye’s fermented food ecosystem.

## Data Availability

No new data were created or analyzed in this study. Data sharing is not applicable to this article.

## Supplementary Materials

The following supporting information can be downloaded at https://www.mdpi.com/article/10.3390/foods14244324/s1, Table S1: PRISMA 2020 Checklist.
